# How did mental health become so biomedical? The progressive erosion of social determinants in historical psychiatric admission registers

**DOI:** 10.1177/0957154X20968522

**Published:** 2020-11-03

**Authors:** Fritz Handerer, Peter Kinderman, Carsten Timmermann, Sara J Tai

**Affiliations:** University of Liverpool, UK; University of Liverpool, UK; University of Manchester, UK; University of Manchester, UK

**Keywords:** Admission registers, biomedical model, psychosocial model, record-keeping, social determinants

## Abstract

This paper explores the historical developments of admission registers of psychiatric asylums and hospitals in England and Wales between 1845 and 1950, with illustrative examples (principally from the archives of the Rainhill Asylum, UK). Standardized admission registers have been mandatory elements of the mental health legislative framework since 1845, and procedural changes illustrate the development from what, today, we would characterize as a predominantly psychosocial understanding of mental health problems towards primarily biomedical explanations. Over time, emphasis shifts from the social determinants of admission to an asylum to the diagnosis of an illness requiring treatment in hospital. We discuss the implications of this progressive historical diminution of the social determinants of mental health for current debates in mental health care.

## Introduction

This article documents a collaboration between clinical psychologists and a historian of medicine. It arose from a comparison of today’s mental health care records with records from the nineteenth century in Liverpool. Current records are criticized for lacking information about the social determinants of individuals’ experiences of mental health problems (Allsopp and Kinderman, 2017; Torres et al., 2017) and focusing instead on diagnoses, with a marked biomedical emphasis (Deacon, 2013; Mayes and Horwitz, 2005). Patient registers from Rainhill Asylum (Merseyside, UK) from 1845, accessed through the Liverpool Archive, revealed a different situation. Comments on the social circumstances in which an individual developed mental health problems were prominent in these records, with an emphasis on psychosocial phenomena. The discrepancy between the two record systems (contemporary and historical) raised questions about the nature and timescale of any changes, and their historical, political, social and professional context. Thus, our aim was to study a long-term historical shift in the structure and contents of asylum admission records in England and Wales to gain a better understanding of the disappearance of statements about the social determinants in the diagnosis of mental illness. We argue that the diminution of this social context information at the health records level is part of a broader conceptual shift and has not primarily been driven by increases in knowledge, but also by economic, political and power interests.

Bureaucratic tools such as patient admission registers shape medical knowledge by structuring information and by highlighting or privileging some data and side-lining others (Hess and Mendelsohn, 2010). At the same time, medical and scientific knowledge affects all forms of medical bureaucracy. Record-keeping is a cornerstone of modern health care (Craig, 1991; Hess and Ledebur, 2011). Record systems reflect and shape diagnostic practice, the establishment of normative judgements, referral patterns, communication between professionals, education, research, and the planning and commissioning of services (Craig, 1989; Mellett, 1981). These impacts appear to extend beyond mental health care, and may have an influence on societies, as diagnoses are used as tools in a range of social functions, from welfare benefits and employment to criminal justice (Dong, 2015). Diagnoses can therefore be understood as ‘boundary objects’, as they impinge on areas outside their origins in clinical care and beyond their actual time of usage (Bowker and Star, 1999).

Patient registers are also important historical materials, first-hand sources, contextualizing current medical and social approaches to mental health care (Risse and Warner, 1992). It is therefore surprising that, to our knowledge, no systematic research on changes in the format and contents of standardized psychiatry admission registers in England and Wales exists. One dissertation deals with general patient registers of England and Canada between 1850 and 1950, but psychiatry registers appear only marginally (Craig, 1989). The present paper attempts an analysis of the development of statutory admission registers, with spotlights on 1845, 1906, 1913, 1930 and 1950, as a path to understanding admission practices and disease concepts today.

In UK law, primary legislation is accompanied by a raft of regulations and policies governing the practical enactment of the legal precepts. These can offer more precise insights into the nature of the systems governing mental health care and their changes over time. In particular, policies and regulations can shed light on the changing relationship between broader social and more strictly medical perspectives.

## Comprehensive social context

Several pieces of legislation in the eighteenth and nineteenth centuries attempted to address the suffering of both poor people and those in madhouses and asylums. This was part of a broader set of reforms, which cemented the role of the state in these fields and in medicine and health more broadly. These pieces of legislation addressed fears about wrongful confinement and regulated the provision of asylum care, making it mandatory for each county in England and Wales to provide such care and creating the regulatory, institutional and conceptual framework in which asylums operated, enmeshed with and built on the local structures of the New Poor Law (the Poor Law Amendment Act of 1834). The Madhouses Act 1774 first addressed the inadequacy of the previous legislative framework in this area by empowering a Committee of the Royal College of Physicians to grant licences to premises housing ‘lunatics’ in London; Justices of the Peace were given these powers elsewhere in England and Wales. The law was reformed in minor ways in 1828 and 1832 before the passage of the Lunacy Act 1845 and the County Asylums Act 1845.

These Acts operated together to provide the legal and physical infrastructure for the care of ‘lunatics, idiots and persons of unsound mind’. In the early years of the nineteenth century, there was increasing concern over the care of people in workhouses or overcrowded asylums (Bartlett, 1999). The controlling institution prior to 1845, the London-based Metropolitan Commissioners in Lunacy, was powerless against unsuitable asylums. The County Asylum Act compelled each county to provide an asylum for ‘pauper lunatics’, who were moved from workhouses into the new institutions. In addition, the Commission of Lunacy was established to monitor asylums, their admissions, treatments and discharges. The Commissioners (three medical practitioners, three lawyers and five laypeople) inspected the asylums of England and Wales by visiting, and also examining casebooks, admission registers, etc.

**Figure 1.**
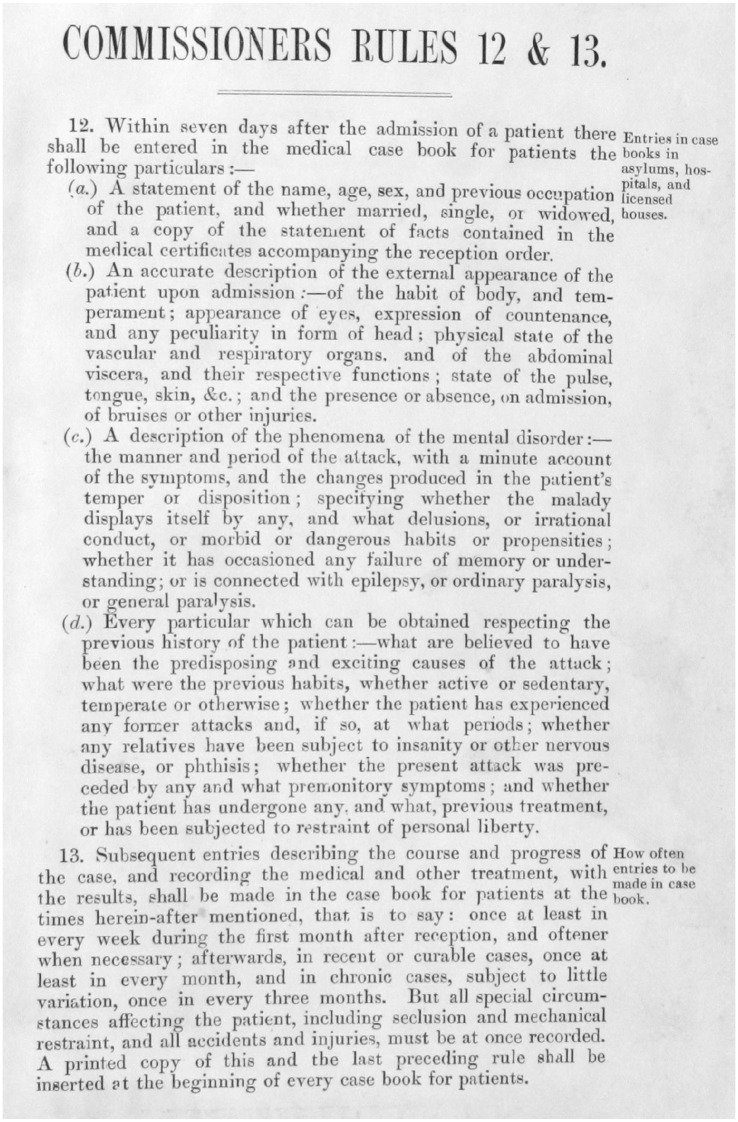
Rules 12 and 13 of the 1846 ‘Rules of the Commission of Lunacy’; from: M614 RAI/1/1 Admission records (inside front cover) for Rainhill Asylum, 1853. © Liverpool Record Office, Liverpool Libraries.

The Commission of Lunacy introduced a high level of procedural reform. Central were the newly established ‘Rules of the Commission of Lunacy’ set out in a circular of 1846.^1^ These Rules included systems of admission, and also the requirement to paste a copy of the relevant sections into the ledgers used for the recording of the details of admission (see Figure 1). Admission of ‘paupers’ required one medical certificate and an Order by a Justice of the Peace, whereas for private patients two medical certificates and an application by a family member were sufficient (the logic being that family members would protect the interests of their loved ones, and everyone could recognize ‘lunacy’). Significantly for this paper, admission registers were introduced, in which an entry by the proprietor or superintendent was required within seven days after reception of a patient (specified in paragraph 50 of the Luncacy Act, 1845); there were penalties for failing to complete an entry or ‘willingly and knowingly’ making untrue notes.

**Figures 2a (above) and 2b (opposite).**
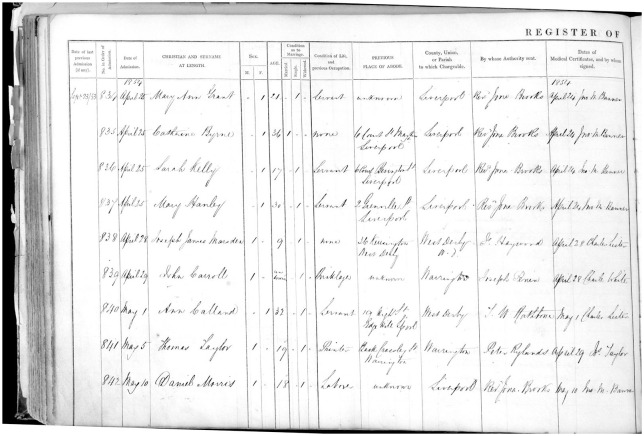
Sample admission register from the Rainhill Asylum 1853 (following the 1845 format); from: M614 RAI/1/1 (p95) Admission records for Rainhill Asylum, 1853. © Liverpool Record Office, Liverpool Libraries.

The form of admission register established by the 1846 Rules consisted of a two-page broadsheet ledger inscribed into columns (see Figure 2) and was (remarkably) used until 1906 in England and Wales.

**Figure:**
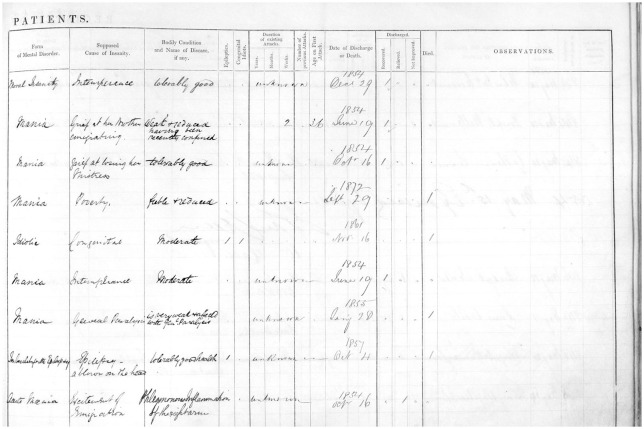


These recorded administrative data (date of admission, dates of previous admission (if any), date of medical certificate, patient number, local authority (for invoicing purposes) and ‘by whose authority sent’), sociodemographic (name, sex, age, marital status, ‘condition of life and previous occupation’, address), diagnostic (form of mental disorder, bodily condition, epileptics, congenital idiots), explanatory (supposed cause), phenomenological data (duration, number, age on first attacks), information on outcome (date of discharge, discharge status [recovered, relieved, not improved, or death]), and observations.

Some earlier publications have noted the particular nature of these admission registers and other kinds of mandatory book-keeping, and their potential for offering insights into the nineteenth-century view of mental health problems (Bartlett, 1999; Scull, 1993). Bartlett commented on the casebooks, which offered more space for observational records (three full pages per person) but presented the same fields of interest. As he puts it:

Even at the point of admission, the casebooks are striking in their focus on the individual, not on diagnosis or the characteristics of the mental disease of the patient. With the exception of heredity, a factor which the medical superintendent was specifically instructed to watch for by the commissioners’ direction, all other major causes of insanity listed by the case books reflect social conditions of the individual. Insanity was caused by grief, intemperance, childbirth, religion, or poverty, according to the case books. (p. 218)

This pattern was also clear in our analysis of the ‘supposed cause of insanity’ in the Rainhill Asylum Admission Register from 1852 to 1855. Rainhill Asylum had opened as the Third Lancashire County Lunatic Asylum on 1 January 1851, and became the County Lunatic Asylum, Rainhill in 1861.^2^ Notably, the hospital was the location of the Great Porridge Strike of 1913, when the staff, who were members of the National Asylum Workers’ Union, went on strike in protest at the replacement of meat with oatmeal porridge.

In 1852, a total of 242 male and female patients were admitted to the Rainhill Asylum, diagnosed with a form of ‘mania’, ‘melancholia’ or ‘dementia’. Interestingly, not every patient got a diagnosis. The most common cause recorded (with 78 cases) was ‘unknown’ (and it should be noted that the Rules did not permit the column to be left empty, but an entry of ‘unknown’ was acceptable). ‘Intemperance’ was frequently reported (*n* = 24), as was ‘childbirth’ and ‘stillbirth’ (*n* = 9), and ‘poverty’ (*n* = 8). Single examples from the following years demonstrate the generally unlimited potential of the cause column: ‘domestic unhappiness, wife kissing with another man’, ‘loss of child by drowning’, or ‘injury to head by a fool’.^3^

In the week of 21 March 1853, the ‘causes’ recorded in the admission register included ‘unknown’, ‘domestic distress’, ‘remorse after seduction’, ‘religious excitement’, ‘the deprivations of poverty’, ‘hereditary disposition’, ‘poverty and want of proper training’ and ‘epilepsy’. For example, a woman was admitted on 25 April 1854, with a cause recorded as ‘grief at her brother’s leaving her to emigrate’, and the comment that she ‘is said to talk incessantly about being on board a vessel. Passes sleepless nights and is very incoherent’. Moreover, the comments in the casebook give more details on the woman who lost her child by drowning:

Insanity was brought on in this case by the loss of her child, who accidentally fell into a well near her dwelling and she saw the body pulled out and knowing that it was that of her own child. At first the [affection or affliction] manifested itself as hysterical which later however she has become subject to fits of violence and occasionally has refused her food.

By 27 January 1855, the notes record that she had refused food, and on the next day she refused to drink. She died at 5 p.m. on the 28 January 1855.

One significant implication is that these admission registers and casebooks (and the operation of the Act) were built on a commonly comprehensible understanding of mental health problems. The majority of the Commissioners in Lunacy were not medically trained. In the view of the Earl of Shaftesbury, Chair of the Commissioners from 1845 to 1885,

it having been once established that the insanity of a patient did not arise from the state of his bodily health, a man of common sense could give as good an opinion as any medical man he knew [respecting the treatment and the question of his sanity]. (Quoted in Scull, 1993: 211)

In the 1830s, similar patient registers at the Charité, Berlin, were explicitly written so as to be understandable to laypeople (Hess and Ledebur, 2011), and learned journals of that time underline this psychosocial focus; for example, John Hawkes, an Assistant Medical Officer in an English asylum, wrote: ‘intemperance very frequent; distress, in most cases arising from poverty; want, insufficient food, loss of work, anxiety as to maintenance, &c. such are some of the most common causes of insanity in the poor’ (Hawkes, 1857). When the Commissioners in Lunacy recognized an increase of pauper patients, their first attempt to explain this situation was in the psychosocial context, even though unsuccessfully: ‘We are unable to discover any material changes in the social condition of the labouring population rendering them more prone to mental disease.’ (Fifteenth Report of the Commissioners in Lunacy to the Lord Chancellor, 1861).^4^

The aim, and consequence, of the book-keeping bureaucracy and oversight was to bring asylums and madhouses under the control of the Commissioners in Lunacy and hence of the Lord Chancellor. In practical terms, the introduction of mandatory registers, reports, and investigations led to considerable paperwork. That could be observed in the way in which the reports by the Commissioners to the Lord Chancellor grew in length. These annual reports subsumed all reports of the individual asylums. The annual report was a mere 41 pages in 1851, but extended to 241 pages in 1861 and 443 pages in 1879 (Mellet, 1981).

This developing control and oversight led to complaints from medical practitioners. Although sanctions by the Commissioners were in fact limited, medical practitioners complained about a ‘centralised despotism’ (Mellet, 1981: 240). This coincided with the growth of epidemiology and a statistical approach to the understanding of illnesses and treatment, manifested in 1850 with the foundation of the Epidemiological Society, which had been supported (and presided over) by Lord Shaftesbury (White, 1993). In the Commissioners’ report of 1876, improved statistical analysis was described as a solution to ‘the question of insanity in its various aspects’. Columns for ‘supposed causes’ and ‘observations’ left blank for free text entry constituted problems to the statistical approach. Individual explanations for insanity got lost in general statistics. This is visible, for example, in the Annual Report for 1906 to the Commissioners by the Newcastle on Tyne city asylum. All noted causes between 1889 and 1906 had been clustered into 30 categories, partly as abstract as ‘adverse circumstances &c’.

Both the Lunacy Act 1845 and the County Asylums Act 1845 were subsequently replaced by the Lunacy Act 1890. The most important change affected the confinement of private patients, where a ‘legal certificate’ became obligatory, in order to address concern about inappropriate detention. The Lunacy Act of 1890 therefore introduced ‘reception orders’, authorizing detention in asylums; these were issued by a specialized Justice of the Peace, but could be renewed at regular intervals by submission of a medical report to the Lunacy Commission. The admission register remained unchanged.

## Reorganizing the data

Bureaucratic or legislative control of a chaotic and potentially abusive system was a key aim of the Lunacy Act 1890. This ‘triumph of legalism’ (Jones, 1993: 93) led to a significant reduction in the number of private asylums, from 82 in 1889 to 54 in 1930 (Takabayashi, 2017: 251). Since psychiatrists tended to earn much more money in the treatment of private rather than ‘pauper’ patients, this reduction has been called a ‘professional crisis’ (p. 246).

Therefore, following the 1890 Act, pressure continued to reform mental health legislation. Between 1902 and 1905, a statistical subgroup of the Medico-Psychological Association,^5^ led by Dr Percy Smith, developed a new register which would record information in what was considered to be a more appropriate fashion, namely one in which ‘(i)ndividuality must be to a certain extent sunk’ (Smith, 1905: 734). The recommended changes, said to be ‘framed on the teachings of experience by those most competent to the task, may well be considered to fairly embrace the whole field of aetiological inquiry’ (Coupland, 1910: 1).

These ‘teachings of experience’ included the 1859 publication of Charles Darwin’s *On the Origin of Species*, and the research on genetic inheritance by Georg Mendel published in the 1860s and rediscovered as the foundation of the new science of genetics in the early twentieth century. In 1869, Francis Galton’s *Hereditary Genius* was published, and in 1892 August Weismann’s *Das Keimplasma: eine Theorie der Vererbung* (*The Germ Plasm: A Theory of Inheritance*); 1895 saw the discovery of X-rays, opening a completely new view of the human body, literally and metaphorically, as an object of scrutiny.

In part as a result of concerted medical lobbying (Bond, 1902; Jones, 1906), the Commissioners in Lunacy published in 1906 a new set of regulations, based on the recommendations by Dr Percy Smith and his team. What had been the ‘admission register’ was split into the civil register and the medical register to enable a more precise description (Rules of Commissioners in Lunacy, 31 October 1906). In this new system, 53 different aetiological codes were substituted for the narrative record of supposed causes.

In the context of the central issue for this paper, namely the changes to the consideration of social determinants, this apparently technical change facilitated a progressive diminution of the role of social factors and the unique experiences of individuals needing mental health care. Experiences such as ‘domestic unhappiness, wife kissing with another man’ or ‘loss of child by drowning’ would probably be coded as F1 or F2 – sudden or prolonged mental stress. Potentially valuable details such as ‘injury to head by a fool’ would henceforth be recorded simply as I1. Moreover, both the prominence and extent of codes related to ‘heredity’ (A1 to A5), ‘toxic’ (H1 to H10), ‘diseases of the nervous system’ (K1 to K5) and ‘other bodily affections’ (L1 to L8) all illustrate the growing focus on biological factors.

The admission registers of that time reveal a cautious introduction of these new codes. The registers offered a free-text column for the ‘principal’ aetiological factor, and also an extra column for the code schedule. Additionally, ‘(a)s many Contributory Factors as may be found are to be entered’ in another free-text entry column with an associated column for codes.

These changes represent a shift from qualitative to quantitative approaches. While plausible in the pursuit of good governance, causes like ‘poverty’ (which had been the third most frequently coded cause in the Rainhill Admissions Register in 1854, after ‘intemperance’ and ‘unknown’), ‘grief’ or ‘domestic troubles’ (which were among the five most frequently used causes in Newcastle), loss of loved ones, mistreatment or social isolation all disappeared. Instead, reported statistics reflect a strong emphasis on heredity and bodily phenomena. It is also worth noting that (entirely reversing the earlier policies of the Lunacy Act 1890 that such registers should be accessible to the layperson) the format of the register from 1906 onwards made it impossible for most people to understand. A similar development was also apparent at that time in Scotland (Andrews, 1998: 259).

With these new registers and new aetiological codes, statistics relevant to ‘causes and associated factors’ became, as intended, a regular part of the Report to the Lord Chancellor. By 1913, ‘insane heredity’ had become by far the most often coded cause. In the years between 1907 and 1911, an average of 5083 cases were given the ‘A1’ code each year. This result can be seen as an example of ‘paper technology’ (Hess and Mendelsohn, 2014: 22), as it was not knowledge-driven but caused by a specific format of bureaucracy. As a register developed by psychiatrists for psychiatrists, this can also be seen as a consolidation of power, through specialized language, at a time when the profession feared that it was losing authority.

## The influence of empiricism and eugenics

The regulations surrounding the records and registers changed again with the passage of the Mental Deficiency Act 1913. This Act principally addressed the needs of people to whom we would now refer as living with learning disabilities. It was, even at the time, a controversial law.

The ideas in Sir Francis Galton’s *Hereditary Genius* (1869) led in 1907 to the establishment of the Eugenics Education Society, later renamed the Eugenic Society, which elected Galton as their Honorary President until his death in 1911 (Keynes, 1993). This coincided with the invention of IQ tests in 1906 as a psychometric tool that reified the theoretical construct of intelligence. This period also saw an increasing use of photographs in casebooks. Without any change in legislation, from the 1870s to the mid-twentieth century, photographs became a widely used tool (Bhanji, 2002) for physiognomic purposes (Diamond, 1857). Galton had recommended the use of photographs as early as 1879 as a method to recognize insanity, capturing biological phenotypes (Galton, 1879), and psychiatric admission registers started to include photographs from the early years of the twentieth century.

A sign of the power and influence of the young Eugenic Society was the establishment of a Royal Commission on the Care and Control of the Feeble-Minded, to address what were then seen as worries about a ‘defective’ underclass breeding more rapidly than the healthy working-class population (Jackson, 2000: 206). The Commission recommended against compulsory sterilization, despite a strongly worded appendix to its 1908 report^6^ by Galton himself, but a flavour of these discussions can be seen in the comments of Herbert Samuel, MP, who, in a debate in 1905, commented that ‘the incapable ought to be employed on farm colonies, and the unwilling, the wastrels, and tramps ought to be employed on penal farm colonies’ (HC Deb, 1905).

This 1908 report of the Royal Commission on the Care and Control of the Feeble-Minded contained eugenically motivated speculations about the heredity of mental deficiency, and claimed that feeble-mindedness would be genetically linked to crime, pauperism and alcohol (Woodhouse, 1982). Many witnesses were members of the Eugenics Education Society, as was the Commission’s Chair, and the recommendations won important supporters such as Winston Churchill (*The Times*, 16 July 1910).^7^

This formed at least some of the basis for the Mental Deficiency Act 1913, which aimed to provide for the care and management of four classes of people: Idiots, Imbeciles, Feeble-minded persons and Moral Imbeciles; the last were described in the Act itself as displaying ‘mental weakness coupled with strong vicious or criminal propensities, and on whom punishment has little or no deterrent effect’. The powerfully eugenic nature of this legislation was clear at the time. When debated in Parliament, the MP Josiah Wedgwood (voting against the Bill) said ‘It is a spirit of the Horrible Eugenic Society which is setting out to breed up the working class as though they were cattle’ (House of Commons, 1912a).

Although the Mental Deficiency Act 1913 was not primarily targeted at people we would now describe as people living with a mental health problems (and, indeed, neither repealed nor substantially reformed the central provisions of the Lunacy Act 1890), it marked a significant shift in UK mental health legislation. This Act yet again made changes to the bureaucracy and regulatory systems of mental health law. The oversight body became the Board of Control for Lunacy and Mental Deficiency, which gained additional powers. That Board was made up of no more than 15 members (of whom no more than 12 were to be remunerated), at least four of whom were legal members, four medical and one female. In contrast to the Commissioners in Lunacy, it was no longer mandatory to have lay members, and the role of medical practitioners grew. The Act contains strong elements of eugenic thinking. ‘Idiocy, imbecility, feeble-mindedness and moral imbecility’ were defined as conditions present from birth on, or from an early age. People identified as falling into these categories were confined to institutions or to guardianship under the same conditions as the Lunacy Act 1890 (involving, procedurally, two medical certificates and one legal Order). Once in a sex-separated institution (to prevent the detainees from having children; Hide, 2014: 38), patients had to stay for one year, when a report by one medical practitioner decided on their leaving or staying. After the first year, this procedure was repeated every five years. As early as 1912, this had been criticized by a Member of Parliament (Wedgwood again): ‘when you put this autocratic power in the hands of specialists whom we cannot criticise because we do not know on what ground they base their executive action, it is then you are introducing a serious danger to the State’ (House of Commons, 1912b).

In terms of admission registers, the 1913 Act continued to apply some of the rules of the Lunacy Act 1845: the superintendent had to make entries within two days after admission, except of the ‘bodily condition’ and ‘form of mental disorder’, which had to be made within seven days. However, significant changes also appeared. There was, after 1913, no column for ‘supposed causes’, and nothing about ‘duration’, ‘number of attacks’ or ‘age on first attacks’. Instead, a very precise selection of four diagnoses, spelled out in detail on every sheet, was required.

Once again, we see a progressive conceptual and procedural move from a psychosocial understanding, to largely biological and genetic explanations (a differentiation first described by Karl Jaspers in 1913). The clear statutory requirements for comprehensive records of social determinants and narrative causes of individuals’ journeys into the care of the asylums under the 1845 Act had gone. The change in Regulations in 1906 diminished the attention given to social determinants when narrative causes were reduced to a very limited set of unambiguously biomedical aetiological codes, and were further undermined with the passage of the Mental Deficiency Act of 1913, with its emphasis on hereditary and biological determinism, the omission of an explanatory column and its echoes of eugenics.

Although a feature of the ‘casebooks’ rather than admission registers, a visible example of this eugenic thinking is a focus on twins and heredity. By 1913, hospital casebooks included a large section devoted to twins – whether the index patient was a fraternal or identical twin and, in that event, whether or not the twin had ever experienced mental health problems, and the detailed mental health history of maternal and fraternal family back to grandparents.

## Treatment in a classified and confined context

The law was reformed again with the Mental Treatment Act 1930. This, as the name implies, marked a further significant shift away from a social concept of ‘asylum’ towards a medical concept of ‘hospital’ and ‘treatment’.

The early years of the twentieth century saw significant medical advances, including developments in the understanding of the role of chemicals acting as neurotransmitter substances (Otto Loewi won a Nobel Prize in 1923 for the discovery of the role of acetylcholine as an endogenous neurotransmitter), and the equally significant but slightly more controversial ‘malarial therapy’ (a therapeutic intervention based on the notion that the fever induced by malarial infection killed off the syphilis bacterium) which also won its inventor, Julius Wagner-Jauregg, a Nobel Prize and was an indicator of a growing interest in physical and biological treatment of mental illness (Tsay, 2013). Further therapies in that vein appeared, with the first leucotomy surgery conducted in 1935 (this also won its originator the Nobel Prize, in 1949) and electroconvulsive therapy, used for the first time in England in 1939 and considered, though unsuccessfully, for the Nobel Prize (Hansson and Fangerau, 2016). These therapies contrasted with concepts of moral treatment that had dominated since the early nineteenth century (Rollin, 1994). Mental illness, many argued, had to be approached much more like other forms of disease, and described using the categorial nosology approach of Emil Kraepelin (Jablensky, 2007).

There were similarly dramatic developments in the science of psychology. The rise of behaviourism, as a discipline solely concentrating on psychology as measurable phenomena, arguably began when John Watson published ‘Psychology as the Behaviorist Views It’ in 1913. In 1920, he and Rosalie Rayner conducted the ‘Little Albert’ experiment on the classical conditioning of fear (Watson and Rayner, 1920), inspired by Ivan Pavlov’s experiments on conditional reflexes, and in 1923 Sigmund Freud published ‘*The Ego and the Id*’. All these developments, while psychological as well as biomedical, clearly placed emotions and mental health in the domain of science and medicine.

In that context, the Mental Treatment Act 1930 introduced voluntary admissions into all institutions dedicated to the treatment of and care for those considered mentally ill, including public mental hospitals and registered private nursing homes. It furthermore established temporary admission, which made it possible to confine patients for six months against their will, if the psychiatrists thought it appropriate. Neither forms of admission required legal certificates. They have been described as the solution to the professional crisis after 1890 (Takabayashi, 2017). However, these changes also further empowered psychiatrists as medical authorities who could rule on insanity and circumstances in which it is deemed curable. Significantly, the Mental Treatment Act 1930 also coined the term ‘mental hospital’, formalizing this developing link with organic diseases.

**Figure 3.**
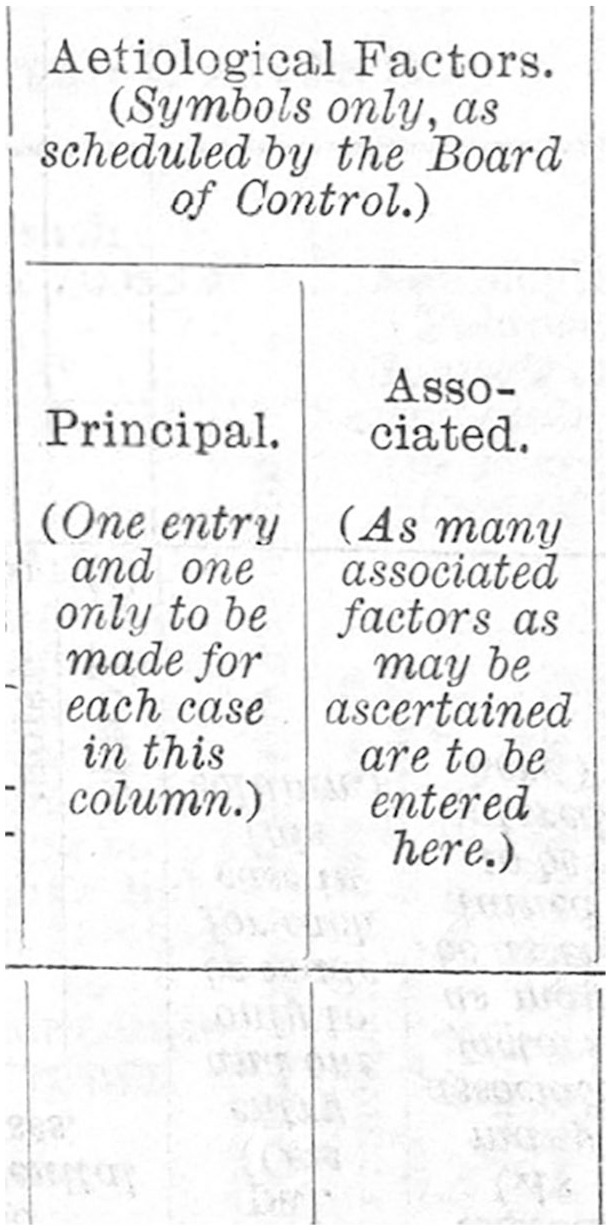
‘Aetiological Factors (symbols only . . .)’ in the admission register following the Mental Treatment Act 1930 and associated Statutory rules and orders, no. 1083, Lunacy and mental treatment, England; from: RET/1/5/5/2/1/32 (p42) Statutory Rules and Orders 1930, No. 1083, Lunacy and Mental Treatment, England. © Borthwick Institute for Archives, the University of York.

Like most previous Acts, the change in the law in 1930 brought changes to the Rules, noted in ‘Statutory rules and orders, no. 1083, Lunacy and mental treatment, England’.^8^ In spite of its length (69 pages), rules regarding the medical register are vague. Entries were required to be made by a medical practitioner within three months, but without penalties for omission. The register also changed, slightly, but in ways that are crucial for our argument. The aetiological-code schedule introduced in 1906 was included unrevised in the new register, whereas the possible entry columns were reduced from four to two, by deleting the free-text spaces. Thus, as can be seen in Figure 3 (note the phrase ‘symbols only’), practitioners had no option other than to use the codes in these small columns, removing the opportunities for free-text entry. Bearing in mind that the codes were introduced in order to facilitate statistics on the aetiological factors of illness, it is relevant to examine the reports to the Lord Chancellor. These reports reveal significantly that the Board of Control received such statistics from the Commissioners in Lunacy only in 1914 and 1921. In all other years between 1914 and 1945 ‘causes’ were solely mentioned in the context of death, and, after 1930, statistics of laboratory findings were common (for the rising importance of laboratory science, see Sturdy and Cooter, 1998).

## Nationalized medical services

The establishment of the UK’s National Health Service in 1948 transferred the Board of Control to the Ministry of Health. It also added one medical practitioner to the Board, consistent with the described progressive medicalization of mental health services. There were no regulatory changes regarding admission records at this time, but the introduction of the sixth revision of the World Health Organization’s *International Classification of Diseases and Causes of Death* (ICD) in 1948 did lead to changes. This offered, for the first time, a structured classification for ‘mental, psychoneurotic and personality disorders’, and can be seen as further medicalization of mental health; hand-in-hand with the idea of mental ‘hospitals’ as places for treatment are mental disorders placed in a classification of diseases. There was no specific mental health legislation in England and Wales until 1959, when the Mental Treatment Act 1930 was repealed by the Mental Health Act 1959. This abolished the Board of Control, and provided a legal framework for consensual treatment (for most people receiving mental health care) as well as providing for involuntary admission to hospital. The 1959 Act was subsequently replaced by the Mental Health Act 1983, later revised in 1995, 2001 and 2007.

The use of specifically medical terminology was again tightened up considerably in the Mental Health Act 1959, which stated that it was defining ‘mental disorder’, but defined it only in so far as: ‘In this Act “mental disorder” means mental illness, arrested or incomplete development of mind, psychopathic disorder, and any other disorder or disability of mind; and “mentally disordered” shall be construed accordingly’; this is essentially circular.

It is striking that none of these Acts gave particular instructions on the form of records to be used. The Mental Health Act 1959 stated that the Minister ‘may make regulations for . . . prescribing the form of . . . any document . . . [and] for requiring the manager of hospitals and local health authorities to keep such registers or other records’ (section 50). But the admission registers from the Rainhill hospital reveal an ongoing use of the formula from 1930 between 1946 and 1950. The picture emerging is that – consistent with the transfer of responsibilities to the new National Health Service, and more specifically the local Health Authorities – local medical record-keeping was thought appropriate. In Rainhill, therefore, from 1950 a four-page document replaced the earlier two-page registers. This included results of ‘Blood Chemistry’, ‘Cerebro-Spinal Fluid’ and ‘Urine’, as well as detailed questions about twins (‘not a twin, triplet, etc./ twin, same sex/ twin, different sex/ twin, sex unknown/ triplet, etc./ not known if a twin, triplet’ . . . ‘has other twin been dealt with under L. & M.T. Acts?’).

Most interestingly for the purpose of this paper is that, after 1950, aetiological factors no longer appeared in the records. After a constant reduction over years, the psychosocial circumstances of poor mental health had not only been reduced to ‘aetiological factors’ but had by now disappeared completely. It seems as if the ‘form of mental disorder’, the diagnosis, grew (figurative and literally) into the ‘cause’ of itself (corresponding to the circular definition mentioned above). An English survey from 1955 ends with the conclusion: ‘It is felt that valid information about the influence of sociological factors upon the form and incidence of mental disorders must await a more exhaustive process of fact-finding than is possible in a survey based upon existing hospital records.’ (Carstairs, Tonge, O’Connor and Barber, 1955).

Record-keeping finally shifted from the registers of admission, established under the Lunacy Act, to hospital records. This remains the position to the present day, a situation itself the subject of considerable controversy, specifically in respect to the ways in which social determinants are recorded (Kinderman and Allsopp, 2018).

## Conclusion

This paper explores the history of the statutory record-keeping in relation to the recognition of social determinants of mental health. By examining statute law, associated Rules and Regulations, and illustrative examples of completed admissions registers located in local authority archives (principally the records of the Rainhill Asylum, located at Liverpool Archive), it has been possible to explore the changing ways in which mental health systems have used and recorded information related to social context and causal issues in mental health. The fact that statute law and regulations apply and are enforced throughout the UK means that we have thorough, and more or less complete, information across the nation and through time.

This analysis reveals a clear progressive diminution of the role of social determinants in reporting on the development of mental health problems, and presumably in understanding and responding to distress. The registers from 1845, with their free-text column for the ‘presumed causes’, led to entries primarily related to the psychosocial contexts of patients. Progressive amendments to the Rainhill admission registers in 1906, 1930 and 1950 reveal a distinctive development of a biomedical focus.

Of course, in the nineteenth century and today, those offering mental health care know a great deal of detail about the lives and circumstances of those seeking help. We must presume that staff are aware of the stories of their patients’ lives, although we should also note that psychiatric care is routinely criticized as still highly medicalized, with little human contact, and is frequently even brutalizing.^9^ But now, as it was in the past, only a tiny proportion of the information available to nurses and psychiatrists (even psychologists) is made available to the official, national statistics. This is, in truth, the point: what information, from all that is potentially available, is made public, is formally and universally recorded, and is used to form the basis of policy-making?

We need statistics to plan care, to decide on the provision of staff and physical infrastructure; we can see how difficult it is to derive useful data from narrative accounts. However, biomedical diagnoses have been widely criticized for their unscientific nature and poor internal consistency and utility (Allsopp, Read, Corcoran and Kinderman, 2019). The heterogenous and invalid nature of these diagnoses leaves current statisticians poorly armed. The ICD in its newest, 11th, version offers a promising alternative, by providing codes for particular psychological phenomena and psychosocial context factors (Allsopp and Kinderman, 2017; Kinderman and Allsopp, 2018). Knowledge about social determinants of health is vital for effective and equitable health-care policy-making (Fisher and Baum, 2010).

This historical analysis has revealed how, over time, we have lost the individual stories that inform services about social determinants of mental health problems. Whereas admission registers in 1845 describe a woman who was destroyed by the sight of her dead son being dragged from a well, by 1930 officials were instructed to use only one code (F2) from an approved list. The history tracks a path of progressive diminution of the social determinants of mental health, perhaps contextualizing the current position where social determinants are almost completely absent from such records (Kinderman and Allsopp, 2018).

## Notes

1. Extracts of these rules can be found in the Merseyside Record Office: Admission register 1851–1856 (Admission numbers: 1–1128): Catalogue M614 RAI/5/1.

2. The Asylum records are held by the Merseyside Record Office, located at Liverpool’s Central Library.

3. Merseyside Record Office: Admission register 1851–1856 (Admission numbers: 1-1128): Catalogue M614 RAI/5/1.

4. Available from the Wellcome Trust collection: https://wellcomelibrary.org/collections/browse/collections/digasylum/

5. A body first established in 1841 as the ‘Association of Medical Officers of Asylums and Hospitals for the Insane’; it changed its name to the Royal College of Psychiatrists in 1971.

6. Report of the Royal Commission on the Care and Control of the Feeble-Minded; accessed (8 Sep. 2020) at: https://wellcomelibrary.org/item/b28038551

7. Cited in: Reporting on the work of the Royal Commission on the Care and Control of the Feeble-Minded; accessed (8 Sep. 2020) at: https://www.thetimes.co.uk/archive/page/1910-07-16/8.html

8. Accessed (8 Sep. 2020) at: https://wellcomelibrary.org/item/b24956533

9. An example was accessed (8 Sep. 2020) at: https://www.bbc.co.uk/news/uk-england-41914555
